# Assessing the Relationship between Neurocognitive Performance and Brain Volume in Chronic Moderate–Severe Traumatic Brain Injury

**DOI:** 10.3389/fneur.2016.00029

**Published:** 2016-03-10

**Authors:** Nikos Konstantinou, Eva Pettemeridou, Ioannis Seimenis, Eleni Eracleous, Savvas S. Papacostas, Andrew C. Papanicolaou, Fofi Constantinidou

**Affiliations:** ^1^Center for Applied Neuroscience, University of Cyprus, Nicosia, Cyprus; ^2^Department of Psychology, University of Cyprus, Nicosia, Cyprus; ^3^Department of Medical Physics, Medical School, Democritus University of Thrace, Alexandroupolis, Greece; ^4^Medical Diagnostic Center “Ayios Therissos”, Nicosia, Cyprus; ^5^Neurology Clinic B, The Cyprus Institute of Neurology and Genetics, The Cyprus School of Molecular Medicine, Nicosia, Cyprus; ^6^Division of Clinical Neurosciences, Department of Pediatrics, The Le Bonheur Neuroscience Institute, University of Tennessee Health Science Center, Memphis, TN, USA; ^7^Division of Clinical Neurosciences, Department of Neurobiology and Anatomy, The Le Bonheur Neuroscience Institute, University of Tennessee Health Science Center, Memphis, TN, USA

**Keywords:** TBI, VBM, grey matter, white matter, neurocognitive/neuropsychological assessment, brain atrophy

## Abstract

**Objectives:**

Characterize the scale and pattern of long-term atrophy in gray matter (GM), white matter (WM), and cerebrospinal fluid (CSF) in chronic moderate–severe traumatic brain injury (TBI) and its relationship to neurocognitive outcomes.

**Participants:**

The TBI group consisted of 17 males with primary diagnosis of moderate–severe closed head injury. Participants had not received any systematic, post-acute rehabilitation and were recruited on average 8.36 years post-injury. The control group consisted of 15 males matched on age and education.

**Main measures:**

Neurocognitive battery included widely used tests of verbal memory, visual memory, executive functioning, and attention/organization. GM, WM, and CSF volumes were calculated from segmented T1-weighted anatomical MR images. Voxel-based morphometry was employed to identify brain regions with differences in GM and WM between TBI and control groups.

**Results:**

Chronic TBI results in significant neurocognitive impairments, and significant loss of GM and WM volume, and significant increase in CSF volume. Brain atrophy is not widespread, but it is rather distributed in a fronto-thalamic network. The extent of volume loss is predictive of performance on the neurocognitive tests.

**Conclusion:**

Significant brain atrophy and associated neurocognitive impairments during the chronic stages of TBI support the notion that TBI results in a chronic condition with lifelong implications.

## Introduction

Traumatic brain injury (TBI) has been historically considered as a single event that requires rehabilitation during the months after the injury and results in a static course thereafter (1). However, an emerging hypothesis views TBI as a long-term condition with chronic and possibly progressive consequences rather than a static condition following a short recovery phase (1–4). Neuropsychological research indicates that following an acute recovery phase many patients with moderate–severe TBI sustain significant cognitive deficits lasting for many years post-injury (5–8). Epidemiological evidence suggests that a single moderate–severe TBI is the strongest acquired risk factor for developing dementia later in life (9, 10).

During the acute and subacute stages of TBI, Wallerian degeneration, inflammation, apoptosis, excitotoxicity, and prolonged hypo-perfusion result in white matter (WM) and gray matter (GM) volume loss (11–13). Neuroimaging studies demonstrated that these effects extend beyond the first year post-injury and individuals with TBI exhibit significant brain volume loss in both the subacute and chronic phases (3, 4, 14–18) that continues for years after the injury (3, 5, 15, 19). For example, recent reports demonstrated GM and WM volume loss for up to approximately 4 years post-injury (17).

The reported brain atrophy resulting from GM and WM volume loss could potentially explain the neurocognitive deficits observed in moderate–severe TBI many years post-injury. However, previous studies that assessed brain volume loss focused primarily on either mild TBI cases [e.g., Ref. (20, 21)] or on moderate–severe TBI during the acute/subacute recovery phase and only up to approximately 4 years post-injury (3, 5, 17, 22). Moreover, studies that have examined brain atrophy in chronic moderate–severe TBI patients have either not examined the associated neurocognitive deficits or have focused on just a select few neurocognitive measures (17, 23, 24–28). Therefore, it is unclear if the well-established neurocognitive deficits of moderate–severe TBI observed many years post-injury (7, 8) are related to brain atrophy and to what extend. The central aim of this study, therefore, was to begin to shed light on the question of the relationship between brain volume and cognitive outcome during the chronic stages of moderate–severe TBI.

Most previous research that investigated the chronic effect of TBI on brain volume and its relationship with cognitive outcome are complicated by the confounding effects of rehabilitation. Systematic post-injury rehabilitation has been shown to improve cognitive functioning of patients with TBI (6, 29, 30). For example, Till et al. (8) showed that regardless of injury severity, the amount of rehabilitation received at 5 months post-injury was the best predictor of cognitive outcome. It is, therefore, important to gain an understanding of the true chronic effect of TBI on brain volume and its relationship to cognitive outcome: what is the true extent of brain atrophy and how does it relate to cognitive functioning in the absence of systematic post-acute rehabilitation? For instance, if only a subset of TBI patients demonstrates significant brain atrophy during the chronic stages that is related to cognitive deficits, this will direct future research (and clinicians) to assess and evaluate specific risk factors that include type of injury and specific mechanisms (e.g., contusions, diffuse axonal injury, blast, or repeat concussions). On the other hand, if brain atrophy and the associated cognitive deficits are ubiquitous, this will suggest that TBI itself is a degenerative disorder. In order to address this question and gain a more accurate understanding of the true chronic effect of TBI on brain volume and its relationship to cognitive outcome, the present study, therefore, included participants who had not received systematic post-acute comprehensive rehabilitation.

Sex differences in cognitive outcomes present another confounding factor of previous research investigating the chronic effects of TBI on the relationship between brain volume and cognitive outcome. Animal studies of TBI demonstrated better outcomes among females than males. These findings supported the idea that gonadal steroids (e.g., estrogen and progesterone) may have a neuroprotective role after TBI (31–34). However, research on sex differences in humans with TBI is limited and often contradictory (35–37). In order to avoid such possible confounds due to sex differences and due to the higher prevalence of male over female TBI patients (38), the current study investigated a homogeneous group of male participants.

The present study is the first part of a larger project that is aiming to establishing a TBI cohort with moderate–severe TBI in order to investigate the neurophysiological substrates of cognitive deficits associated with the chronic stages of moderate–severe TBI. The project utilizes an array of different designs, such as group comparisons and case studies, using a range of magnetic resonance imaging (MRI) data, including volumetric measures [e.g., voxel-based morphometry (VBM)], diffusion tensor imaging for characterization of WM tracts, and resting-state fMRI for assessing the effects of TBI on brain connectivity. Here, we focused on the relationship between cognitive outcome and brain volume in different types of brain tissue. We also examined the scale and spatial pattern of such brain atrophy by comparing a group of male participants with chronic TBI who were well beyond the spontaneous recovery phase to a group of neurologically healthy males matched on age and education.

First, we calculated percent brain volume change between the control group and the participants with TBI. Next, we examined whether such atrophy is widespread or localized on specific brain areas. We employed VBM for assessing local GM and WM volume at individual locations in the brain and performed statistical comparisons between the participants with TBI and the non-injured controls. Finally, we investigated the relationship between brain volume and cognitive outcome, on a comprehensive set of neurocognitive measures. We employed individual differences analysis to examine whether global measures of brain volume [GM, WM, and cerebrospinal fluid (CSF)] and the volume of regions-of-interest (ROIs) that exhibited volumetric differences between the TBI and the control groups in the VBM analysis hold any predictive value regarding the cognitive outcome of TBI patients. It was hypothesized that participants with moderate–severe TBI would demonstrate significantly reduced whole-brain GM and WM volume and increased CSF as compared to matched non-injured participants. The atrophy would not be widespread, but localized in specific regions. Finally, greater degree of atrophy was expected to be associated with lower performance on neuropsychological measures assessing memory performance, executive functioning, and attention/organization.

## Materials and Methods

### Participants

The participants with TBI were compared to a group of healthy volunteers matched on age, education, and socioeconomic status (all TBI participants except two were pair-matched with participants in the control group). All participants were males. The Cyprus Bioethics Committee approved all study procedures and a consent form was obtained from every participant. Following is a description of each group (see Table 1).

**Table 1:** **Demographic information of the TBI patient group**.

Patient ID	Age (years)	Education (years)	TSI (months)	Mechanism of injury	GOSE
1	37	16	139	Assault	6
2	23	13	63	Motor vehicle accident	6
3	24	13	24	Motorcycle accident	6
4	24	14	84	Pedestrian with vehicle collision	3
5	41	8	36	Fall (work or other non-sports related)	4
6	60	11	156	Motor vehicle accident	5
7	24	15	60	Motorcycle accident	4
8	35	17	27	Fall (work or other non-sports related)	7
9	32	16	60	Motor vehicle accident	6
10	47	12	274	Motor vehicle accident	6
11	30	18	179	Motorcycle accident	6
12	21	12	40	Motorcycle accident	4
13	29	12	166	Fall (work or other non-sports related)	8
14	29	16	24	Object falling	8
15	24	12	72	Motor vehicle accident	3
16	33	12	228	Motor vehicle accident	3
17	29	14	76	Fall (work or other non-sports related)	5
	(*M* = 31.88; SD = 10.04)	(*M* = 13.59; SD = 2.53)	(*M* = 53.38; SD = 74.73)		(M = 5.29; SD = 1.61)

*TSI, time since injury*.

#### Participants with TBI

Nineteen participants with brain injury met the study criteria and were included in the study. Participants were recruited from collaborating physicians using a rolling admission process. Two participants were subsequently excluded from the analysis due to difficulties with the MRI procedures (one participant had claustrophobia and the MR images from the second participant yielded significant motion artifacts rendering the imaging data unusable). The ages of the remaining 17 participants ranged from 21 to 60 years with a mean age of 31.9 years (SD = 10 years). Education ranged from 8 to 18 years, with a mean of 13.6 years (SD = 2.5 years). Participants were recruited on an average 8.36 years post-injury (range = 2–22.8 years, SD = 6.34 years, Mdn = 6 years). None of the TBI participants had sustained blast or repeated concussions. All of the TBI participants had sustained diffuse axonal injury with contusions as evidenced by their MRI scans.

Functional outcome was also assessed during the neurocognitive assessment indicating the presence of significant (moderate–severe) disability several years post-injury. Specifically, Glasgow Outcome Scale Extended (GOSE) analysis indicated the following distribution of recovery: three participants had achieved good recovery (18%), eight were rated with moderate disability (47%), and six with severe disability (35%).

Following are the inclusion/exclusion criteria for participants with TBI, which are consistent with the Constantinidou et al. criteria (6, 39): age between 18 and 60 years; native speaker of the Greek language; primary diagnosis of moderate–severe closed head injury (CHI) at least 12 months prior to the study recruitment. The indication of an initial moderate–severe head injury was determined by the presence of three or more of the following severity indices: (1) initial Glasgow Coma Scale score <12, (2) abnormal initial computed tomography (CT) or MRI findings indicating acute central nervous system pathology, (3) length of impaired consciousness >20 min as specified by the emergency records, (4) length of post-traumatic amnesia >24 h as specified in the acute hospital/emergency records, (5) length of acute hospital stay >3 days, (6) abnormal neurological examination on hospital admission and discharge indicating focal sensory and motor deficits, or changes in mental status attributed to brain injury, (7) medical complications secondary to the injury, and (8) head injury severity classification according to hospital records. Other inclusion/exclusion criteria consisted of the Rancho Los Amigos Scale Level VI or higher (which indicates appropriate goal-oriented behavior, and post-traumatic amnesia resolution); no aphasia present with the exception of mild-to-moderate word-finding problems due to cognitive deficits.

Participants were excluded if they had a penetrating head injury, if they were diagnosed with stroke at the time of injury, if they had a premorbid central nervous system disorder or learning disability, if they had a premorbid major depression or other significant psychiatric disorder as defined by the Diagnostic and Statistical Manual of Mental Disorders (40), and if they had an active or current alcohol, drug, or other controlled substance abuse that would interfere with participation in the study.

Primary causes of TBI were consistent with those reported in industrialized nations (22, 38): 50% of the participants were injured in motor vehicle accidents and another 25% were injured as a result of work-related falls. The remaining 25% were injured as a result of assaults, falling of objects, and pedestrian–vehicle collision. None of the participants had received systematic and comprehensive post-acute comprehensive rehabilitation in the past or at the time of study recruitment. Some of the participants received inpatient rehabilitation services and fragmented individualized outpatient treatment during the acute phase of their recovery. All participants were residing at home at the time of study participation.

#### Control Group

Sixteen non-injured males were recruited for the study. These participants were volunteers from the greater Nicosia and Limassol areas who met the study’s inclusion/exclusion criteria. One participant was unable to complete the MRI examination due to claustrophobia and was subsequently excluded from the present analysis. All of the remaining participants were Greek-speaking adults ages 21–60 years (M = 33.8 years, SD = 10.3 years) with no history of a neurological condition or brain trauma, documented psychiatric history, learning disability, or substance abuse. Education ranged from 8 to 18 years (M = 13.4 years, SD = 2.6 years).

### Tests and Materials

The testing protocol consisted of the MRI acquisition, neurocognitive, and experimental testing protocols. The entire battery of neurocognitive tests and experimental tasks lasted approximately 2 h and was administered over two sessions. Testing included scheduled breaks in order to avoid mental fatigue of participants.

#### Neurocognitive Tests

The neurocognitive battery included widely used tests of verbal memory, visual memory, executive functions, and attention/organization.

The verbal memory test battery included the Greek adaptation of the Auditory Verbal Learning Test [total score in trials 1–5, difference score between trial 5 and trial 1, short delay free recall, long delay free recall, and list A true positive recognition score (41)], the Digit Span Forward and Backwards total score [adapted Wechsler Memory Scale-Revised (WMS-R)] (42), the adapted paragraphs from the WMS-R Logical Memory I and II free recall subtests (sum of the score and the sum of the delay recall).

The visual memory test battery included the Rey Complex Figure Test [immediate recall, delayed recall, recognition total score (43)], the Visual Span Forward and Backwards [adapted from WMS-R (42)], the spatial visual short-term memory (VSTM) experimental task threshold, and the object VSTM experimental task threshold (see task description below).

The executive functions tests battery included the Symbol Digits Modalities Test (ref), the Trail Making Tests A and B (44), and the phonological (letter F) and category recall (Animal recall) from the Control Oral Word Association Test [COWAT; (45)].

Tests of attention/organization included the Rey Complex Figure Test [copy and time to copy (43)], as well as the Distractibility index and the mean reaction time (RT) in the experimental response competition task (see description below). Outcome measures were also obtained in terms of the GOSE (46).

#### Experimental Tasks

The experimental tasks were controlled using the Cogent Toolbox^1^ for Matlab (MathWorks, Inc.) on a Lenovo PC running Microsoft Windows 7 attached to a 15”CRT monitor (60-Hz refresh rate).

Participants performed a response competition task [e.g., Ref. (47)] for assessing both speed of processing and distractibility for each participant, as well as a delayed match-to-sample task for assessing VSTM capacity separately for objects and for spatial locations.

**Figure 1:**
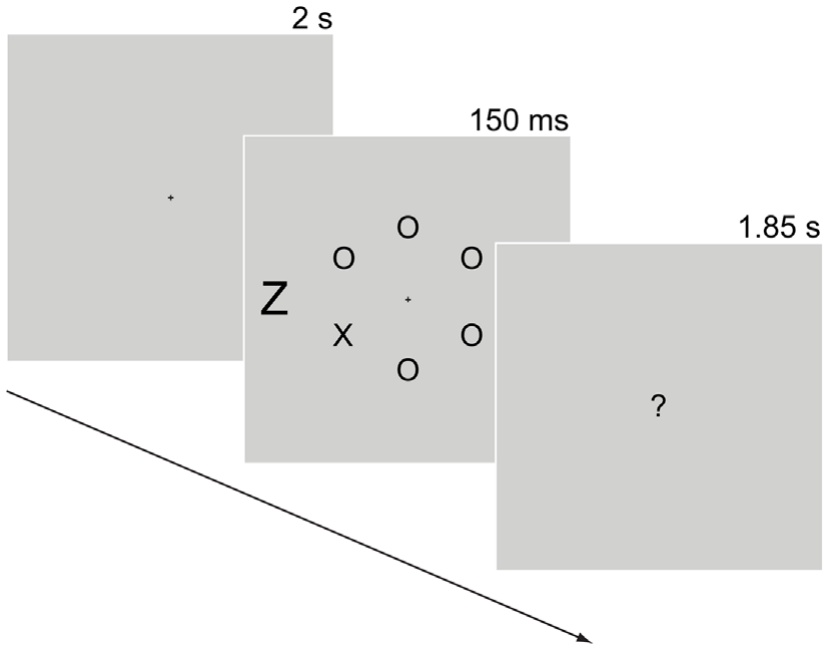
**Response competition task**. Trials started with a 2-s fixation cross. Next, six letters appeared in a circular arrangement (2° in radius) containing one of two target letters (“X” or “Z” subtending 0.6°× 0.4°) and five non-target letters (all Os). Participants searched for the target letter among the non-target letters. The target letter was equally likely to appear on any of the six positions of the circle. A distractor-letter (subtending 1°× 0.6°) that was equally likely to be congruent (e.g., distractor “X” when target was “X”) or incongruent (distractor “Z” when the target was “X”) with the target letter appeared 3.5° to the left or to the right of the fixation point. A display with “?” at the center appeared after the stimulus display for 1.85 s during which participants responded to the target letter by pressing 0 for “X” or 2 for “Z” using the numerical keypad. An auditory tone (“beep”) was used as feedback for incorrect responses. An example trial sequence in the incongruent condition is shown here. Display durations appear above each display. Stimuli are not drawn to scale.

The response competition task required participants to make speeded responses to a target letter in the presence of a peripheral distractor that was either congruent (same as target letter e.g., distractor “X” when the target was an “X”) or incongruent (e.g., distractor “Z” when the target was an “X”; see Figure 1). Slower RTs to the target letter in the incongruent versus the congruent condition indicated a failure to ignore the distractor letter (48). Each participant’s mean RT in trials with a correct response (correct identification of the target letter) and the distractibility index (the mean RT difference between correct congruent and correct incongruent trials) were used in the constructed measure of attention/organization.

**Figure 2:**
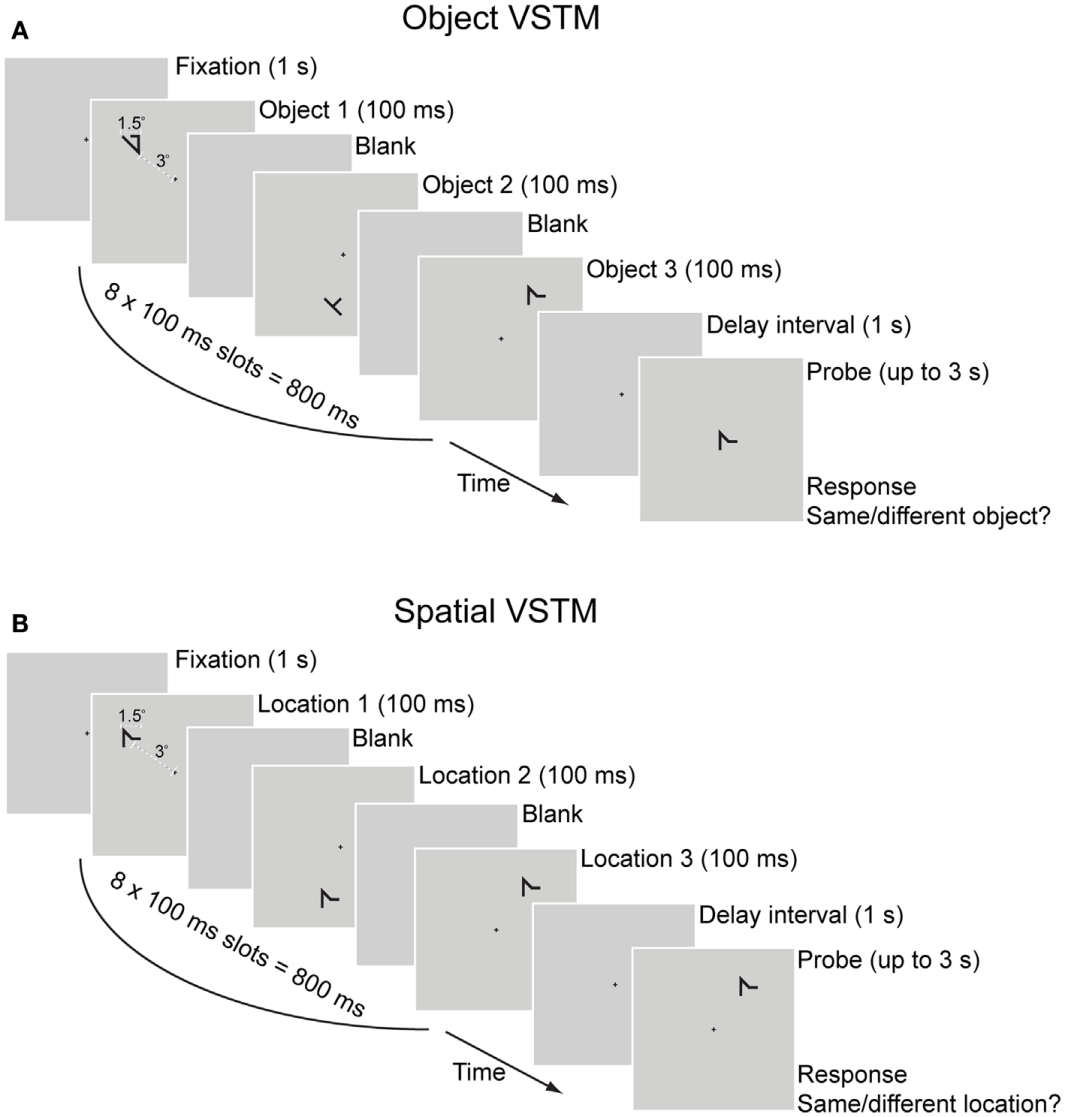
**Visual short-term memory task**. Each trial had a duration of up to 5.8 s. Trials started with fixation cross for 1 s, followed by eight sample displays with a duration of 100 ms each (total sample displays duration was 800 ms), a blank delay interval (1 s), and a test display/response period (up to 3 s). An auditory tone (“beep”) was used as feedback for incorrect responses. Each item in the memory-set appeared in one of the eight sample displays in random order (e.g., in a trial with a set size of three items shown here, each of the three items was randomly assigned to one of the eight sample displays). Participants were instructed to maintain the memory-set items in VSTM throughout the retention interval by visually projecting them on the screen while avoid verbalizing them. During the response period, participants pressed a button to respond whether the memory probe item was of the same identity as any of the memory-set items **(A)** or it appeared at the same location as any of the memory-set items **(B)**. In order to minimize load on object VSTM, all items in the spatial memory-set task were of the same identity. Responses were made on a standard QWERTY keyboard with the right index and middle fingers pressing the numeric keypad buttons 1 for “same” and 2 for “different”, respectively. The memory probe was a match on half of the trials, and appeared at a different location (for the spatial VSTM task) or was of a different shape (for the object VSTM task) on the other half.

Participants also performed a delayed match-to-sample VSTM task, as shown in Figure 2, maintaining a set of shapes in VSTM throughout a 1-s retention interval by visually projecting them on the screen while avoiding verbalizing them. During the response period of the task, participants pressed a button to respond whether the memory probe item appeared at the same location as any of the memory-set items for the spatial VSTM task or whether the memory probe item appearing at fixation was of the same identity as any of the memory-set items. This design enabled us to assess separately the two distinct mechanisms of spatial and object VSTM (49, 50). In order to discourage participants from verbalizing the shapes used in the memory set, these were drawn from a pool of meaningless symbols that are difficult to verbalize [see Ref. (48, 51, 52)], for similar manipulations of VSTM load. Using these items of irregular shapes in a task of very rapid presentation (100 ms each) allowed participants very little time for verbalizing, thus ensuring that the task taxed visual memory instead of verbal memory resources.

Each participant’s VSTM capacity was calculated using a 3-up/1-down staircase. Specifically, participants started with a practice block of 12 trials used to familiarize them with the task. Following practice, each participant completed a block of trials for each of the object and the spatial VSTM tasks that contained a memory set of two items in the first trial. For consecutive trials, the size of the memory set depended on the response given in the previous trials. The number of items in the memory set was increased by one item after three consecutive correct responses or decreased by one item after an incorrect response. The staircase was terminated after 10 reversals, with a reversal defined as any change in the direction of the staircase. Individual VSTM capacity estimates were calculated as the mean memory-set size at each of the last eight staircase reversals.^2^

#### Standard Score Transformation

Scores from the full set of neurocognitive and experimental tests were combined into four composite scores representing the conceptually motivated constructs of verbal memory, visual memory, executive functions, and attention/organization. Specifically, each participant’s score on each of the individual measures was transformed into a standard score (*z*-score) using the following method: first, each participant’s score on the individual measures was subtracted from the mean score of the healthy control group on the corresponding measure and divided by the SD of the control group. Next, each standard score was balanced such that higher scores indicated better performance. The resulting individual standard scores (*z*-scores) were then averaged together to derive an individual score for each constructed measure.

#### Image Acquisition

MR images were acquired with a 3.0-T scanner (Achieva, Philips Medical Systems, Best, The Netherlands). The built-in quadrature RF body coil and a phased array 8-channel head coil were used for proton excitation and signal detection, respectively. An isotropic, three-dimensional (3D), T1-weighted rapid acquisition gradient-echo sequence (fast field echo; repetition time = 25 ms; echo time = 1.85 ms; flip angle = 30°) was utilized to acquire whole brain, transverse MR images with an acquisition/reconstruction voxel of 1.0 mm × 1.0 mm × 1.0 mm (data interpolation was not implemented in any direction to improve resolution and reduce partial volume effects). The scanning session also included other standard pulse sequences (e.g., T2-weighted turbo spin echo, diffusion weighted imaging, and fluid-attenuated inversion recovery) to exclude significant brain pathology of a different etiology.

#### Volumetry

We hypothesized that there would be overall group differences in the volume of GM, WM, and CSF. Specifically, we expected that compared to the control group, the TBI group would demonstrate significant GM and WM volume loss but significant increase in CSF volume.

Individual brain volume calculation was performed using the Individual Brain Atlases Statistical Parametric Mapping toolbox [IBASPM; (53)] under MATLAB 8.1 (MathWorks, Natick, MA, USA). IBASPM uses the segmentation routines of SPM5 (Wellcome Department of Cognitive Neurology, Institute of Neurology, University College London, London, UK). The MR images were segmented into GM, WM, and CSF and individual volumes for each tissue type were then extracted. Percent volume change between the control group and the TBI group was calculated using the formula (mean control group volume − mean TBI group volume)/(mean control group volume) × 100. This index allows quantification of tissue volumetric changes between the two matched groups.

#### Voxel-Based Morphometry Pre-Processing and Analysis

We also hypothesized that the TBI group would demonstrate volume reduction in specific brain regions, manifested as significant differences in a VBM comparison.

Pre-processing steps for VBM were performed using SPM8 and included segmentation of the MR images into GM and WM, followed by a Diffeomorphic Anatomical Registration Through Exponentiated Lie Algebra (DARTEL) for inter-subject registration of the GM and WM images (54, 55). During this co-registration pre-processing, local GM and WM volumes were conserved by modulating the image intensity of each voxel by the Jacobian determinants of the deformation fields computed by DARTEL. The registered images were smoothed with a Gaussian kernel (full width at half maximum = 8 mm) and were then transformed to Montreal Neurological Institute (MNI) stereotactic space using affine and non-linear spatial normalization implemented in SPM8 for statistical comparisons.

The pre-processed images were entered into two-samples *t*-test models in SPM5. A statistical threshold of *p* < 0.05, corrected for the whole-brain volume at a cluster level using the “Non-Stationary Cluster Extent Correction” toolbox for SPM5^3^ (56), was used as an indicator of regions with significant differences in GM volume or WM volume between the TBI and the healthy control groups. The design matrix included the study group (TBI and control) and the age and years of education of the participant as covariates of no-interest. Since all participants were males, gender was not included in the design matrix.

## Results

### Group Comparisons

#### Demographics

The TBI and the control groups were very similar in terms of age and education, as expected [age, *t*(30) = 0.238, *p* = 0.814; education, *t*(30) = 0.544, *p* = 0.590; two-tailed two-samples *t*-tests]. Any significant differences in subsequent comparisons cannot, thus, be attributed to sample differences in terms of age or education.

#### Neurocognitive Performance

Pairwise comparisons were conducted in order to compare the performance of the two groups on the constructed measures of verbal memory, visual memory, executive functions, and attention/organization. As shown in Table 2, compared to the non-injured control participants, the performance of participants with TBI was significantly lower on all four constructed measures, indicating significant neurocognitive impairment at several years post-TBI.

**Table 2:** **Performance on neurocognitive constructed measures**.

Measure	TBI group	Control group	Statistics		
	
	Mean (SD)		*t*	df	*p*	Cohen’s *d*
Verbal memory	−0.35 (0.85)	0.41 (0.60)	2.90	30	0.001	0.92
Visual memory	−0.27 (0.71)	0.29 (0.46)	2.61	30	0.007	0.85
Executive functions	−0.39 (0.85)	0.44 (0.35)	3.48	30	0.001	1.05
Attention/organization	−0.21 (0.56)	0.23 (0.30)	2.73	30	0.005	0.88

*df, degrees of freedom; *p*, one-tailed*.

#### Volumetry

**Figure 3:**
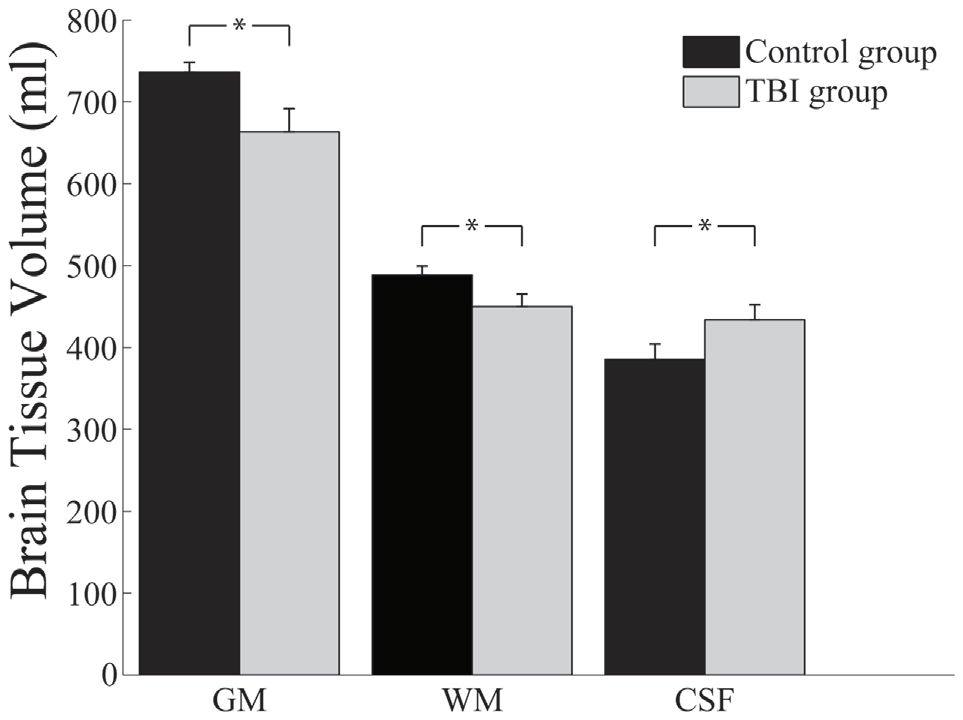
**Brain tissue volumes (milliliters) as a function of participant group and brain tissue type**. GM, grey matter. WM, white matter. CSF, cerebrospinal fluid; *p = 0.05.

As shown in Figure 3, mean GM volume was significantly reduced in the TBI group (M = 666 ml, SD = 50 ml) compared to the control group (M = 736 ml, SD = 103 ml), *t*(30) = 2.51, *p* = 0.018, Cohen’s *d* = 0.82.

White matter was also found to be significantly reduced in the TBI group (*M* = 454 ml, SD = 43 ml) compared to the control group (*M* = 489 ml, SD = 56 ml), *t*(30) = 1.96, *p* = 0.03 (one-tailed), Cohen’s *d* = 0.66. In contrast to the consistent reduction of both GM and WM volumes, the TBI group exhibited higher CSF volume (*M* = 440 ml, SD = 76 ml) compared to the control group (*M* = 385 ml, SD = 67 ml), *t*(30) = 2.16, *p* = 0.039, Cohen’s *d* = 0.72.

#### Voxel-Based Morphometry

**Figure 4:**
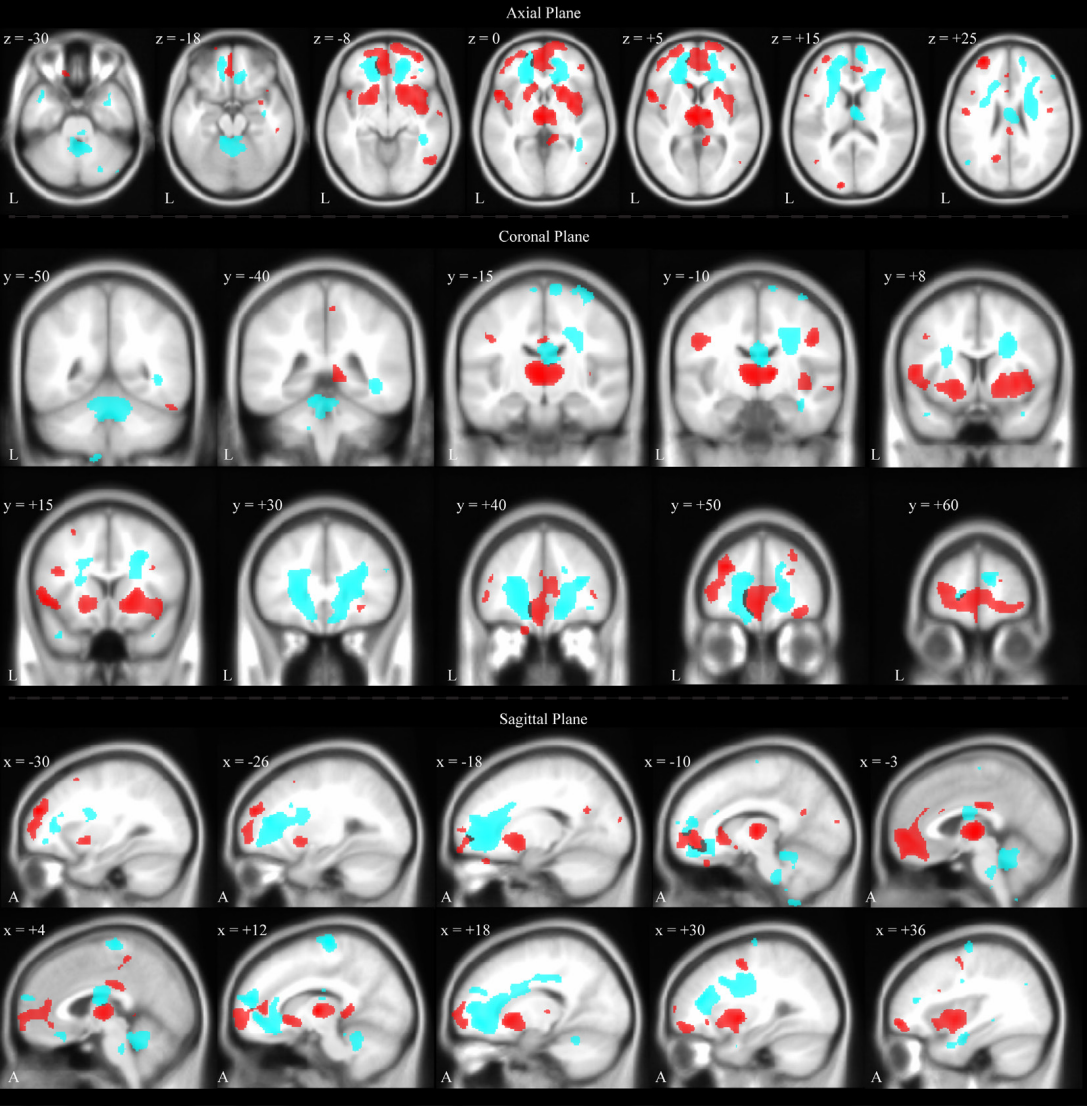
**Brain areas with significant volume reduction in the TBI group compared to the Control group overlaid on an MNI template brain**. Gray matter is color-coded red. White matter is color-coded cyan. Results are overlaid onto the MNI152 T1 standard template. Axial views on first row, coronal views on second and third rows, sagittal views on fourth and fifth rows. L, left. A, anterior.

Voxel-based morphometry analysis was used to identify brain regions with significant GM and WM volume reduction in the TBI group compared to the control group. As shown in Table 3, reduced GM volume in the TBI group compared to the control was found in orbitofrontal cortex in a large coherent cluster extending over the superior and middle orbital gyrus, left and right thalamus, left and right temporal lobe, left inferior frontal gyrus, left and right putamen, and left and right insula (see also Figure 4). No brain areas were found with significantly greater GM volume in the TBI compared to the control group.

**Table 3:** **Brain regions with significantly less gray matter and white matter volume in the TBI compared to control group and their Pearson product-moment correlations with neurocognitive measures**.

	Anatomical region	Tissue type	Side	MNI coordinates			Peak-*z*	Verbal memory	Visual memory	Executive functions	Attention
				*x*	*y*	*z*					
1	Middle frontal gyrus	GM	L	−30	53	25	4.49	0.26	0.13	−0.07	0.08
2	Middle orbital gyrus	GM	L	−2	53	−3	4.06	0.54**	0.40*	0.31*	0.38*
3	Middle orbital gyrus	GM	R	36	56	−8	3.93	0.30*	0.18	0.16	0.24
4	Thalamus	GM	L	−3	−15	3	4.47	0.58**	0.48**	0.63**	0.55**
5	Thalamus	GM	R	12	−10	4	4.35	0.45**	0.29	0.52**	0.34**
6	Temporal pole	GM	L	−52	14	−2	4.34	0.16	0.26	0.03	−0.04
7	Insula	GM	L	−45	15	−6	3.97	0.30*	0.39*	0.13	0.17
8	Putamen	GM	L	−18	8	−5	3.78	0.62**	0.60**	0.57**	0.53**
9	Putamen	GM	R	32	2	−3	4.27	0.44**	0.34*	0.35*	0.35*
10	Putamen	GM	R	18	15	−3	4.08	0.62**	0.61**	0.48**	0.51**
11	Insula/temporal pole	GM	R	45	12	−8	3.91	0.36*	0.17	0.14	0.25
12	Superior medial	WM	R	9	56	15	4.19	0.47**	0.38*	0.33*	0.29
13	Midle orbital	WM	R	14	48	−3	4.07	0.38*	0.34*	0.39*	0.22
14	Middle frontal	WM	R	30	36	16	3.96	0.24	0.22	0.24	0.12
15	Cerebellum	WM	L	−9	−42	−17	3.99	0.17	0.10	0.38*	0.14
16	Cerebellum	WM	R	4	−48	−30	3.87	0.41*	0.43**	0.56**	0.38*
17	Cerebellum	WM	R	3	−52	−18	3.47	0.32*	0.28	0.43**	0.32*
18	Middle frontal	WM	L	−26	38	13	3.86	0.51**	0.53**	0.49**	0.39*
19	Middle frontal	WM	L	−18	39	−0	3.82	0.58**	0.58**	0.56**	0.45**
20	Anterior cingulate	WM	L	−10	48	13	3.79	0.62**	0.59**	0.51**	0.43**

*GM, gray matter. WM, white matter. L, left. R, right*.

**n* = 32*.

*Significant at the 0.05 level (one-tailed).

**Significant at the 0.01 level (one-tailed).

Table 3 also shows brain areas with reduced WM volume in the TBI group compared to the control group (see also Figure 4). Brain areas with reduced WM volume in the TBI group (compared to the control group) were found in bilateral frontal cortex, the thalamus, and the cerebellum.

### Individual-Differences Analysis

Pearson product-moment correlation analysis was employed for the individual differences analysis in order to investigate the relationship of GM, WM, and CSF volume with variables of interest.

#### Relationship between Tissue Volumes

First, we sought to examine whether there was a relationship between the volumes of GM, WM, and CSF. An initial analysis that included all participants from both groups revealed a significant positive correlation between GM and WM volume, *r*(32) = 0.64, *p* < 0.001. CSF did not exhibit a significant relationship with either GM, *r*(32) = −0.04, *p* = 0.830, or with WM, *r*(32) = 0.04, *p* = 0.826.

However, when the analysis was repeated separately for each group, for the TBI group none of the comparisons exhibited significant relationships (all *r* < 0.12). The same comparisons for the control group showed a significant positive correlation between GM volume and WM volume, *r*(15) = 0.80, *p* < 0.001. CSF volume was unrelated to the volume of GM or WM in the Control group, GM, *r*(15) = 0.27, *p* = 0.33, and WM, *r*(15) = 0.26, *p* = 0.349.

This finding indicates that, in neurologically healthy individuals, there is a close positive relationship between the volume of GM and WM, whereby greater volume of GM is associated with greater volume of WM. However, TBI seems to affect GM and WM in such a way that their volumetric relationship is abolished.

#### Neurocognitive Performance

Next, we sought to examine the relationship of GM, WM, and CSF volume with neurocognitive performance, as assessed by the constructed measures of verbal memory, visual memory, executive functions, and attention/organization.

As shown in Table 4, both GM and WM volume exhibited a significant positive correlation with all four neurocognitive measures, whereby greater volume was associated with higher performance. By contrast, CSF exhibited a significant negative correlation with verbal memory and visual memory where higher CSF volume was associated with lower performance (a trend for a negative correlation with the constructed measures of executive functions and attention/organization did not reach statistical significance, see Table 4).

**Table 4:** **Pearson product-moment correlations of brain volume with neurocognitive measures**.

Neurocognitive measure	Gray matter	White matter	CSF
Verbal memory	0.47**	0.57**	−0.34*
Visual memory	0.51**	0.39*	−0.34*
Executive functions	0.34*	0.46**	−0.23 (*p* = 0.10)
Attention/organization	0.41*	0.49**	−0.28 (*p* = 0.058)

*CSF, cerebrospinal fluid*.

**n* = 32*.

*Significant at the 0.05 level (one-tailed).

**Significant at the 0.01 level (one-tailed).

In order to further investigate the relationship between brain volume and neurocognitive performance, we investigated whether brain regions that exhibited significant differences between the TBI and the control groups in the VBM analysis hold any predictive value for neurocognitive performance. Individual normalized brain volume values from those regions were extracted in 6-mm diameter spherical ROIs from each participant.

The results of this analysis are presented in Table 3. Given the number of comparisons per ROI, the α level was lowered to 0.01 in order to avoid a Type I error. Of the 20 ROIs that exhibited significant volumetric differences between the TBI and the control groups, some may hold significant predictive value regarding the performance of the participants in the neurocognitive measures. All significant correlations were positive, indicating that higher volume was predictive of better neurocognitive performance. Specifically, bilateral thalamus and putamen exhibited a significant correlation with performance in all four neurocognitive measures, indicating that higher GM volume in these ROIs is associated with better performance. Left middle frontal gyrus (lMFG) exhibited a significant correlation with verbal memory. Of the nine WM ROIs, left Frontal areas were significantly correlated with all four neurocognitive measures, whereas the right Frontal areas exhibited a relevant trend but did not reach statistical significance.

This finding demonstrates that the volume of brain regions that exhibit significant differences between TBI and control participants are predictive of neurocognitive performance. Moreover, ROI correlations with neurocognitive performance indicate that the correlations between whole-brain tissue volume (GM and WM) and neurocognitive performance are perhaps driven by focal differences in these ROIs.

#### Functional Outcome

A significant correlation between GOSE scores and WM volume, as shown in Table 5, indicates that higher WM volume is predictive of better functional outcome. No significant correlations were observed between GOSE scores and either GM volume or CSF volume. This finding, taken together with the finding that whole-brain GM volume and whole-brain WM volume are significantly reduced in the TBI compared to the control group, suggests that functional outcome, as measured by GOSE, is more closely related to whole-brain WM rather than GM volume.

**Table 5:** **Pearson product-moment correlations of brain volume with time since injury and functional outcome**.

	Gray matter	White matter	CSF
Time since injury	0.18	0.19	0.66**
GOSE	−0.03	0.51*	−0.11

*CSF, cerebrospinal fluid; GOSE, Glasgow Outcome Scale-Extended*.

**n* = 16. The data of one participant with outlier CSF values were excluded from this analysis*.

*Significant at the 0.05 level (two-tailed).

**Significant at the 0.01 level (two-tailed).

Correlations with ROIs indicated that functional outcome of the TBI participants is also related to volume of subcortical structures. Specifically, significant positive correlations were found with the volume of the thalamus [left, *r*(17) = 0.63, *p* < 0.01; right, *r*(17) = 0.57, *p* = 0.01] and the putamen [left, *r*(17) = 0.56, *p* = 0.01; right, *r*(17) = 0.41, *p* = 0.02].

#### Time Since Injury

A significant positive correlation between CSF volume [calculated using IBASPM; (53)] and time since injury indicated that CSF volume is increased with increasing time since injury, *r*(17) = 0.40, *p* = 0.05. By contrast, whole-brain GM and WM volume did not exhibit a significant relationship with time since injury, GM, *r*(17) = 0.18, *p* = 0.24, WM, *r*(17) = 0.09, *p* = 0.36. No significant correlations were found between the volume of ROIs and time since injury.

## Discussion

The current study investigated the chronic outcomes of ­moderate–severe TBI in a homogeneous group of male survivors of TBI that had not received any systematic post-acute rehabilitation and were recruited several years post-injury.

### Group Comparisons

Participants with TBI, when compared to a matched group of neurologically healthy participants, exhibited significant cognitive deficits on measures of verbal memory, visual memory, executive functioning, and attention/organization. They also exhibited substantial reduction in both GM and WM volumes. Specifically, GM volume was reduced by a mean of 9.60% and WM volume was reduced by a mean of 7.04% in the TBI group compared to the control group. In contrast to the reduction of GM and WM volumes, the TBI group exhibited higher CSF volume by 14.29% compared to the control group. These results extend previous findings by demonstrating the true scale of brain volume loss in a homogeneous group of male TBI participants that were examined several years post-injury and had not received any systematic neurocognitive post-acute rehabilitation. The substantial volumetric differences between the TBI and the control groups suggest that the injured brain remains vulnerable to the effects of the injury for many years following the initial insult (57). Taken together with recent evidence demonstrating that brain atrophy is a significant predictor of dementia (58), these findings have significant clinical implications and can inform treatment and rehabilitation of TBI.

Our findings are consistent with a plethora of previous evidence on the extent of brain atrophy in the acute and subacute phases of TBI (5, 7, 14, 16, 17, 19, 59–67). Several studies that have used structural brain imaging in individuals with TBI for assessing brain atrophy employed a longitudinal design (data were collected at more than one point in time) and showed consistent effects of brain volume loss over several months post-injury (2, 14, 16, 20, 21, 28, 65, 67–69). However, to the best of our knowledge, no outer limit has yet been set on the time period during which the brain atrophies due to the TBI (64). The present findings indicate that significant brain atrophy is evident many years post-injury, but future research needs to determine the rate of TBI-induced atrophy and its relationship to the aging process.

Previous research has suggested that reduction of brain volume in the acute and subacute phases may reflect either resolution of edema or the development of brain atrophy (3, 28). Our findings of significant brain volume loss for many years post-injury clearly show that the existence of brain atrophy long after the injury and after acute or subacute pathophysiology has been resolved. As such, our findings complement these studies and contribute to our understanding of the progression of atrophy by demonstrating the true extent of brain volume loss for many years post-injury in individuals with TBI who had not received any systematic post-acute rehabilitation. These findings support the hypothesis that TBI is the initiation of a chronic disease with long-lasting implications, rather than a single event with a static course (1, 3, 5).

An additional purpose of the study was to characterize the spatial pattern of this atrophy by identifying specific brain regions with significant volume reductions in participants with TBI compared to the control group. Using VBM, such significant volumetric differences were found to be mainly concentrated in a fronto-thalamic network, the cerebellum, and other areas connected to the thalamic network (i.e., putamen and insula). This finding indicates that this brain network is most vulnerable during the chronic stages of TBI.

### Individual Differences

Individual differences analyses indicated that whole-brain GM and WM volume may hold predictive information regarding the level of neurocognitive functioning in verbal and visual memory, executive functioning, and attention/organization abilities. This finding in a group of survivors tested many years post-injury is consistent with previous reports that greater whole-brain GM and WM volume is associated with better neurocognitive performance at up to 4 years post-injury (57).

The volume of CSF also exhibited negative correlations with the neurocognitive measures of visual memory and verbal memory. We note that due to the opportunistic nature of CSF, such correlations may be due to either GM or WM volume loss. However, because both GM and WM loss may be related to verbal and visual memory deficits, correlations of CSF volume with neurocognitive measures need to be interpreted with caution.

In addition to whole-brain correlations with neurocognitive measures, region-specific correlations indicated that poorer performance on all neurocognitive measures was associated with GM volume loss in the thalamus and the putamen, as well as WM loss in left frontal areas. Although the subcortical location of the thalamus is thought to provide some protection from direct injury (70), in chronic TBI significant volume loss has been observed as a result of damaged afferent–efferent connections (28). Due to the widespread and diffuse network of these afferent and efferent connections of the thalamus, even slight atrophy has the potential to disrupt large neuronal networks with significant and widespread cognitive and behavioral implications, as is the case in our study.

The current study also provides evidence of significant brain volume atrophy in the cerebellum and other areas connected to the thalamus (i.e., frontal areas, putamen, insula). Previous research demonstrated that anatomical connections exist between frontal and thalamic areas (71–73), linking atrophy in fronto-thalamic networks to widespread cognitive impairments, including memory and executive functioning (74). Furthermore, our findings demonstrate that WM volume and the volume of the thalamus are correlated to the functional outcome of ­moderate–severe TBI as measured by GOSE. Future work should aim to further investigate the potential connection between the thalamus and WM shown here to be associated with functional outcome.

Interestingly, significant correlations were also observed between time since injury and CSF volume, but no relationship was found between time since injury and either whole-brain or ROI GM and WM volume. Taken together, these findings indicate that although CSF volume is very sensitive to time since injury, time since injury alone cannot predict GM and WM volume in line with previous research showing that GM and WM volume in TBI is affected by a complex combination of other factors (3, 5, 14–18).

Individual differences analysis was also employed to examine the relationship between whole-brain GM and WM volume revealing a strong positive correlation between these two types of brain tissue in the non-injured brain. However, this strong relationship between the volumes of GM and WM is not observed in the TBI brain. This finding, when taken together with the finding that both GM and WM volumes in the TBI brain are reduced, suggests that perhaps the rate of atrophy, due to TBI, is different for GM and WM. Indeed, starting in the first hours following an injury, a gradual diffused degeneration of WM has been observed (75) without related damage to GM (76–78). Our findings support this interpretation of different rates of atrophy, demonstrating a difference in the mean percent volume reduction of GM versus WM in the TBI group, compared to the matched control group (i.e., TBI volume reduction: GM = 9.60% and WM = 7.04%). It should be noted, however, that the aforementioned interpretation should be viewed with caution. An array of possible interpretations for this finding exists (79, 80) and further research is essential to understand the mechanisms behind it, which is beyond the scope of the present study. For example, GM volume consists of neural cell bodies, together with their dendrites, local ramifications of axons, glial cells, and blood vessels; WM consists mostly of bundles of axons (81). It is, therefore, possible that the relationship between the volume of neural cell bodies and their axons remains unaffected by TBI but the relationship between GM and WM volume is abolished in MRI-based volumetric calculations because TBI affects other types of tissue present in GM (e.g., glial cells, blood vessels, etc.). Future research should specifically aim to test this interpretation in a longitudinal design where participants with TBI are tested at different time points in order to assess the rate of atrophy for each tissue type. Such an understanding is necessary for developing treatment and rehabilitation protocols to counteract brain atrophy.

### Conclusion, Limitations, and Future Research

The findings reported in the current study support the hypothesis that moderate–severe TBI results in significant brain atrophy that lingers several years post-injury. While the acute biomechanics of the injury and associated neurobiological cascade may cause diffuse axonal injury, the observed volume reductions in GM and WM during the chronic phases of the injury are clustered in the fronto-thalamic network with associated neurocognitive deficits. One of the advantages of the current study is the ability to capture the true effects of the injury in the absence of systematic post acute comprehensive rehabilitation. Due to the limited rehabilitation services in Cyprus, our TBI participants had not received any systematic residential post-acute neurocognitive rehabilitation or any post-acute outpatient services. However, the current study design does not allow for assessment of the progression of brain atrophy and for this reason, future studies should follow patients prospectively and longitudinally in order to link MRI findings and neurocognitive changes across time and contribute to the growing body of literature aiming at developing predictor models of recovery. The development of predictor models based on MRI and clinical biomarkers could also shed some light on the link between significant TBI and pathological aging associated with high incidence neurodegenerative conditions, such as Alzheimer’s and Parkinson’s disease.

## Author Contributions

NK recruited participants, designed experiments, collected and analyzed data, and wrote the manuscript. EP recruited participants, collected, and analyzed data. IS collected MRI data. EE collected MRI data. SP recruited participants and advised on the manuscript. AP advised on the manuscript. FC recruited participants, designed experiments, analyzed data, and wrote the manuscript.

## Conflict of Interest Statement

The authors declare no conflicts of interest, including financial, consultant, institutional, and other relationships, that might lead to bias or any other conflict of interest.

## Footnotes

^1^http://www.vislab.ucl.ac.uk/cogent.php

^2^The first two trials of the staircase were treated as practice trials and omitted from the threshold calculation. However, the results pattern and significance did not change when we included the first two trials in the threshold calculation.

^3^http://fmri.wfubmc.edu/cms/NS-General
